# A Fast and Powerful Empirical Bayes Method for Genome-Wide Association Studies

**DOI:** 10.3390/ani9060305

**Published:** 2019-05-31

**Authors:** Tianpeng Chang, Julong Wei, Mang Liang, Bingxing An, Xiaoqiao Wang, Bo Zhu, Lingyang Xu, Lupei Zhang, Xue Gao, Yan Chen, Junya Li, Huijiang Gao

**Affiliations:** 1 Institute of Animal Science, Chinese Academy of Agricultural Sciences, Beijing 100193, China; changtianpeng@126.com (T.C.); liangmangbj@126.com (M.L.); ABX2HF@126.com (B.A.); longmao7@live.com (X.W.); zhubo525@126.com (B.Z.); xulingyang@caas.cn (L.X.); zhanglupei@caas.cn (L.Z.); gaoxue76@126.com (X.G.); chenyan0204@163.com (Y.C.); 2 College of Animal Science and Technology, Nanjing Agricultural University, Nanjing 210095, China; weijulong128@sina.com

**Keywords:** empirical Bayes, genome-wide association study, kinship matrix, linear mixed model, proximal contamination

## Abstract

**Simple Summary:**

Improving statistical power and computational efficiency are always the research foci in genome-wide association studies (GWAS). In this study, we proposed a fast empirical Bayes GWAS method, which is based on the linear mixed model framework. The method is called Fast-EB-LMM in short. Results from simulation studies show that the Fast-EB-LMM has the highest power for quantitative trait nucleotides (QTNs) detection, the highest computational efficiency, and the strongest robustness, as compared with the efficient mixed model association (EMMA) and empirical Bayes (EB). Application to beef cattle population also verified the effectiveness of this method. We believe that Fast-EB-LMM is a valuable additional tool for GWAS.

**Abstract:**

Linear mixed model (LMM) is an efficient method for GWAS. There are numerous forms of LMM-based GWAS methods. However, improving statistical power and computing efficiency have always been the research hotspots of the LMM-based GWAS methods. Here, we proposed a fast empirical Bayes method, which is based on linear mixed models. We call it Fast-EB-LMM in short. The novelty of this method is that it uses a modified kinship matrix accounting for individual relatedness to avoid competition between the locus of interest and its counterpart in the polygene. This property has increased statistical power. We adopted two special algorithms to ease the computational burden: Eigenvalue decomposition and Woodbury matrix identity. Simulation studies showed that Fast-EB-LMM has significantly increased statistical power of marker detection and improved computational efficiency compared with two widely used GWAS methods, EMMA and EB. Real data analyses for two carcass traits in a Chinese Simmental beef cattle population showed that the significant single-nucleotide polymorphisms (SNPs) and candidate genes identified by Fast-EB-LMM are highly consistent with results of previous studies. We therefore believe that the Fast-EB-LMM method is a reliable and efficient method for GWAS.

## 1. Introduction

Genome wide association studies (GWAS) have been carried out in humans, plants, livestock, and other species to identify statistical associations between single-nucleotide polymorphisms (SNPs) and complex traits over the past decades [1]. Meanwhile, a variety of GWAS methods and software packages have been developed. With the development of the next generation and the third generation sequencing technologies, we can genotype tens of millions of SNP markers easily. With such a high-density marker map, there are many challenges we have to face in terms of developing efficient statistical methods and computational algorithms.

Among the various GWAS methods, linear mixed models (LMM) are the most efficient in terms of correcting various forms of confounding, such as population structure and cryptic relatedness [2,3]. Since LMM was first applied in GWAS [4], many improved LMM-based GWAS methods have been developed, e.g., the efficient mixed model association (EMMA), the genome wide efficient mixed model association (GEMMA), and the factored spectrally transformed linear mixed model (FaST-LMM). The EMMA method utilizes a spectral decomposition algorithm to build the likelihood function, which is much easier to evaluate and can thus handle large samples and a large number of markers [5]. The GEMMA method is an improved method of EMMA and can have computational speed orders of magnitude faster than EMMA [6,7]. The FaST-LMM method also improves the computational speed by choosing a selected subset of markers to capture the polygenic background effects [8]. These methods are called exact methods because the polygenic variance is re-estimated for each SNP scanned [6,9]. In contrast to the exact methods, several approximate methods have been developed by fixing the polygenic variance at the estimated value under the null model, including genomewide rapid association using mixed model and regression (GRAMMAR), efficient mixed model association expedited (EMMAX), and population parameters previously determined (P3D) [2,10,11]. The approximate methods have increased the computational speed without compromising the statistical power of marker detection. However, no matter what algorithms are used, most LMM-based GWAS methods use a genetic relationship matrix (GRM) to account for unequal relatedness among individuals [12]. The GRM is also called kinship matrix, which is constructed from all markers on the genome [13,14]. Recent studies have shown that inclusion of the candidate marker in the kinship matrix can lead to loss in statistical power, and the decreased power is due to double-fitting of the candidate marker in the model, one is a fixed effect tested for association and the other is a random effect included in the polygene captured by the kinship matrix [3,15]. Listgarten et al. referred to this phenomenon as “proximal contamination” and demonstrated that the LMM with the candidate marker excluded (MLMe) from the kinship matrix has improved the power. This method is called the FaST-LMM method [15]. Yang et al. proposed a MLMe method called genome-wide complex trait analysis leaving-one-chromosome-out (GCTA-LOCO), which uses a kinship matrix constructed from markers of chromosomes other than the one that the scanned marker resides [16]. However, due to the high computational complexity, the LMM-based GWAS methods with the candidate marker included in the kinship matrix is more commonly applied in practice.

In this study, we present a novel LMM-based GWAS method by using a modified kinship matrix to account for the cryptic relatedness. When a candidate SNP is tested, we exclude all SNPs in a 1Mb window around that SNP from the genome and then use all the remaining SNPs to construct the modified kinship matrix. The size of the window is an adjustable parameter for this method. Meanwhile, we use the eigenvalue decomposition and Woodbury matrix identity to ease the computational burden. Here, we consider the SNP effect as the parameter of interest and use the variance component estimated from the null model as a prior variance. We then fit the model using an empirical Bayes method (EB) [9]. We called this method a fast empirical Bayes LMM method (Fast-EB-LMM). To verify the efficiency and reliability of this method, we conduct simulation studies to evaluate the statistical power and false positive rate of the Fast-EB-LMM method using two different simulated datasets. 

## 2. Materials and Methods

### 2.1. Statistical Model for Marker Scanning

We used the standard LMM as the statistical model for marker scan. The LMM automatically controls potential population structure (Q) effects and the polygenic effect via a marker inferred kinship matrix (K). Therefore, it is also called the Q + K model [4]. Let *y* be a vector of phenotypic values for a trait of interest, which is described as:

(1) $$y=X\beta +Z_{k}\gamma_{k}+\xi +e$$

where *X* is a design matrix capturing all non-genetic (fixed) effects, e.g., systematic environmental effects and population structure effects, *β* is a vector of the fixed effects, $Z_{k}$ is a vector of numerical codes for the *k*th marker, e.g., $Z_{jk}=0$ for A_1_A_1_, $Z_{jk}=1$ for A_1_A_2_ and $Z_{jk}=2$ for A_2_A_2_, where A_1_ represents the minor allele while A_2_ represents the major allele, $\gamma_{k}$ is the effect of locus *k* on the trait (treated as a fixed effect), *ξ* is a vector of polygenic (random) effects with an assumed $N\left(0,K{}^{\prime }\phi^{2}\right)$ distribution where $\phi^{2}$ is the polygenic variance and *K*′ is a modified marker inferred kinship matrix (defined later), and *e* is a vector of residual errors with an assumed $N\left(0,I\sigma^{2}\right)$ distribution where $\sigma^{2}$ is the residual error variance.

### 2.2. The Modified Kinship Matrix

The original marker inferred kinship matrix is defined as $K=\frac{1}{d}ZZ^{T}$, where *Z* is genotype indicator matrix of all SNPs and *d* (a normalization factor) is the average value of the diagonal elements of matrix *K*. To avoid “proximal contamination”, we constructed a modified kinship matrix *K*′ by removing the effect of candidate SNP and its flanking SNPs from the original kinship matrix *K*. The *K*′ is defined as follows:

(2) $$K{}^{\prime }=\frac{1}{d}\left(ZZ^{T}-Z_{s}Z_{s}^{T}\right)$$

where *Zs* is a n×s genotype matrix of the s removed SNPs for the *n* individuals.

### 2.3. Parameter Estimation and a Special Algorithm for Fast Computation

(3) y=Xβ+ξ+e

Under the null model, we use the original kinship matrix *K* to account for the polygenic effect. We then have E(y)=Xβ and $Var\;\left(y\right)=K\phi^{2}+I\sigma^{2}$. Let $\lambda =\phi^{2}/\sigma^{2}$, so that $Var\;\left(y\right)=\left(K\lambda +I\right)\sigma^{2}=H\sigma^{2}$. The restricted maximum likelihood (REML) method was used to estimate the variance ratio *λ* under the null model.

We first applied the eigenvalue decomposition algorithm to decompose the *K* matrix, $K=UDU^{T}$, where U is the eigenvectors (an n×n matrix) and $D=\mathrm{diag}\left\{\delta_{1},\ldots ,\delta_{n}\right\}$ is a diagonal matrix for the eigenvalues. The U matrix has a property of $U^{T}=U^{-1}$ so that $UU^{T}=I$. Let $y^{*}=U^{T}y$, $X^{*}=U^{T}X$ and $Z_{k}^{*}=U^{T}Z_{k}$. The transformed null model is:

(4) $$y^{*}=X^{*}\beta +U^{T}\left(\xi +e\right)$$

where $E\left(y^{*}\right)=X{}^{\prime }\beta$ and the variance-covariance matrix of *y* is $Var\;\left(y^{*}\right)=UKU^{T}\phi^{2}+I\sigma^{2}$. Thus, the restricted log likelihood function is:

(5) $$L\left(\theta \right)=-\frac{n-r}{2}\ln\sigma^{2}-\frac{1}{2}\ln\left|H\right|-\frac{1}{2\sigma^{2}}{\left(y^{*}-X^{*}\beta \right)}^{T}H^{-1}\left(y^{*}-X^{*}\beta \right)-\frac{1}{2}\ln\left|X^{*T}H^{-1}X^{*}\right|$$

where $\theta =\left\{\beta ,\lambda ,\sigma^{2}\right\}$ is the parameter vector, *n* is the sample size, *r* is the rank of matrix *X*, H=Kλ+I is the covariance structure. Given *λ*, the maximum likelihood estimates of *β* and *σ*^2^ are

(6) $$\begin{array}{cc} \hat{\beta } & ={\left(X^{*T}H^{-1}X^{*}\right)}^{-1}X^{*T}H^{-1}y^{*}\\ {\hat{\sigma }}^{2} & =\frac{1}{n-r}{\left(y^{*}-X^{*}\hat{\beta }\right)}^{T}H^{-1}\left(y^{*}-X^{*}\hat{\beta }\right) \end{array}$$

Substituting *β* and σ^2^ in Equation (4) by $\hat{\beta }$ and ${\hat{\sigma }}^{2}$ in Equation (5) yields a profiled likelihood function that is only a function of *λ*, as shown below:

(7) $$L\left(\lambda \right)=-\frac{1}{2}\ln\left|H\right|-\frac{1}{2}\ln\left|X^{*T}H^{-1}X^{*}\right|-\frac{n-r}{2}\ln\left(y^{*T}Py^{*}\right)$$

where $P=H^{-1}-H^{-1}X^{*}{\left(X^{*T}H^{-1}X^{*}\right)}^{-1}X^{*T}H^{-1}$. Further investigation of Equation (3) shows that the profiled restricted log likelihood function only requires the log determinant of matrix *H* and various quadratic forms involving *H*^−1^. Now, let us rewrite matrix *H* by:

(8) $$H=K\lambda +I=UDU^{T}\lambda +I=U\left(D\lambda +I\right)U^{T}$$

The determinant of *H* is:

(9) $$\left|H\right|=\left|U\left(D\lambda +I\right)U^{T}\right|=\left|D\lambda +I\right|\left|UU^{T}\right|=\left|D\lambda +I\right|$$

where |Dλ+I| is a diagonal matrix. Therefore, the log determinant of matrix *H* is:

(10) $$\ln\left|H\right|=\overset{n}{\underset{j=1}{\sum }}\ln(\delta_{j}\lambda +1)$$

The restricted log likelihood function also involves various quadratic forms in the form of $a^{T}H^{-1}b$, for example, $X^{T}H^{-1}X$, $X^{T}H^{-1}y$ and $y^{T}H^{-1}y$. Using Woodbury matrix identity, we can rewrite the quadratic form by:

(11) $$a^{T}H^{-1}b=a^{T}U{\left(D\lambda +I\right)}^{-1}U^{T}b=a^{*T}{\left(D\lambda +I\right)}^{-1}b^{*}=\sum_{j=1}^{n}a_{j}^{*T}b_{j}^{*}{\left(\delta_{j}\lambda +1\right)}^{-1}$$

where $a^{*}=U^{T}a$ and $b^{*}=U^{T}b$. Note that $a_{j}^{*}$ is the jth element (row) of vector (matrix) $a^{*}$ and $b_{j}^{*}$ is the jth element (row) of vector (matrix) $b^{*}$.

Using eigenvalue decomposition and Woodbury matrix identity, matrix inversion and determinant calculation have been simplified into simple summations, and thus, the computational speed can be substantially improved.

Finally, a numeric solution of *λ* can be found iteratively using the Newton algorithm:

(12) $$\lambda^{\left(t+1\right)}=\lambda^{t}-{\left[\frac{\partial^{2}L\left(\lambda^{\left(t\right)}\right)}{\partial \lambda^{2}}\right]}^{-1}\left[\frac{\partial L\left(\lambda^{\left(t\right)}\right)}{\partial \lambda }\right]$$

The scanned model (1) can also be written as:

(13) $$y=\left[\begin{array}{cc} X & Z_{k} \end{array}\right]\left[\begin{array}{c} \beta \\ \gamma_{k} \end{array}\right]+\xi +e$$

Once the variance component *λ* is estimated, we use the modified kinship matrix *K*′ accounting for polygenic effects in the scan model. As SNPs are scanned one by one, the *K*′ matrix is always changing. Meanwhile, we use the mixed model equations (MME) to get the best linear unbiased estimates (BLUE) of the fixed effects, including β and $\gamma_{k}$ [17].

### 2.4. Hypothesis Test

The Wald test is used to test the null hypothesis $H_{0}:\;\gamma_{k}=0$

(14) $$W_{k}=\frac{{\hat{\gamma }}_{k}^{2}}{var\left({\hat{\gamma }}_{k}\right)}$$

Under the null hypothesis, $W_{k}$ follows approximately a chi-square distribution with one degree of freedom. The corresponding *p*-value is calculated as:

(15) $$p_{k}=1-Pr\left(\chi_{1}^{2}\leq W_{k}\right)$$

The *p*-value also can be converted into a $-\log_{10}\left(p_{k}\right)$ test statistic for presentation. SNPs are tested one at a time and the GWAS is completed after all SNPs are scanned across the genome.

### 2.5. EMMA and EB

EMMA is a single marker scanning GWAS method which also considers marker effects as fixed. The R package for EMMA can be downloaded from http://mouse.cs.ucla.edu/emma/. EB is an empirical Bayes approach implemented by the SAS program and we compiled it into an R program (R Core Team, Vienna, Austria) [18]. 

### 2.6. Simulation Studies

To evaluate the statistical power, the false-positive rate and the computational efficiency of Fast-EB-LMM, we conducted two simulation experiments using the *A. thaliana* data and the 16th QTLMAS workshop data. Each experiment was analyzed using three methods (Fast-EB-LMM, EMMA and EB).

#### 2.6.1. Experiment 1

The first simulation dataset was downloaded from Wen et al. [19]. As described by Wen et al, 10000 SNPs derived from the *A. thaliana* dataset were used to do the simulation experiment and the sample size was 199 [20]. In the first simulation scenario, only six QTNs were simulated and with allele frequencies being 0.30. The heritabilities of the six QTNs were set at 0.10, 0.05, 0.05, 0.15, 0.05 and 0.05, respectively. The average effect was 10, the residual variance was set at 10.0, and the phenotypes were simulated from model $y=1\mu +\sum_{i=1}^{6}Z_{i}\beta_{i}+\varepsilon$, where ε: MVN(0, 10×I). In the second simulation scenario, the phenotypes with additive polygenic backgrounds were simulated. The polygenic effect was simulated from a multivariate normal distribution $\mathrm{MVN}\left(0,\;K\sigma_{g}^{2}\right)$ where $\sigma_{g}^{2}$ is the polygenic variance and *K* is the kinship matrix. Given $\sigma_{g}^{2}=2$, $h_{g}^{2}=0.092$. Other simulation parameters were the same as those in the first simulation scenario. The phenotypes were simulated from model $y=1\mu +\sum_{i=1}^{6}Z_{i}\beta_{i}+\phi +\varepsilon$, where ϕ: MVN(0, 2×K). In the third simulation scenario, the phenotypes with epistatic backgrounds were simulated. Three epistatic QTNs were simulated, each with $\sigma_{epi}^{2}=1.25$ and $h_{epi}^{2}=0.05$. Other simulation parameters were also the same as those in the first simulation scenario. The phenotypes were simulated from model: $y=1\mu +\sum_{i=1}^{6}Z_{i}\beta_{i}+\sum_{j=1}^{3}\left(C_{j}\#D_{j}\right)\beta_{jj}+\varepsilon$, where $\beta_{jj}$ is the epistatic effect and $\left(C_{j}\#D_{j}\right)$ is its incidence coefficient. In all three simulation scenarios, the phenotypes were repeated 1000 times. Details for the three simulations were described by Wen et al. [19].

#### 2.6.2. Experiment 2

The second simulation dataset was downloaded from the 16th QTLMAS workshop and it consisted of 4100 individuals (G0–G4). Each of the following four generations (G1–G4) consisted of 20 males and 1000 females, generated from the previous generation by randomly mating each male with 51 females. There were no overlapping generations. The genome consisted of 5 chromosomes, each of 100 Mb size and carrying 2000 equally distributed SNPs. Three traits were simulated in order to mimic milk yield, fat yield and fat content with the heritabilities set at 0.35, 0.35, and 0.50. Genetic (co)variances were generated from 50 QTLs with pleiotropic effects and the QTL positions were sampled from among the even SNPs. The GWAS was conducted using 3000 individuals, all females belonging to generations G1–G3, where phenotypic values were measured for three traits (yield deviations). Details of the simulation were described by Usai MG et al. [21].

### 2.7. Beef Cattle Data

#### 2.7.1. Ethics Statement

All animals were treated following the guidelines established by the Council of China Animal Welfare. Experimental protocols were approved by the Science Research Department of the Institute of Animal Sciences, Chinese Academy of Agricultural Sciences (CAAS), Beijing, China (approval number: RNL09/07).

#### 2.7.2. Animals and Phenotypes

The population consisted of 1301 Chinese Simmental beef cattle that were born in Ulgai, Xilingol League, Inner Mongolia, China (45° N, 118° E), from 2008–2014. After weaning, the cattle were moved to the Beijing Jinweifuren fattening farm for feedlot finishing under the same feeding and management system. Individuals were measured for growth and developmental traits until they were slaughtered at the age of 16–18 months; all animals were slaughtered at roughly the same time. The beef carcasses were then separated, and carcass traits were measured. We chose carcass weight (CW) and bone weight (BW) as the target traits. Descriptive statistics for these two traits are shown in Table 1.

#### 2.7.3. Genotype Data and Quality Control

All the 1301 cattle were genotyped by Illumina Bovine HD BeadChip (Illumina, CA, USA). The SNP chips were scanned using iScan and analyzed with the Illumina’s Genome Studio software (Illumina, CA, USA). The marker dataset consisting of 777,962 SNPs were imputed using BEAGLE v4.1 (Univ Auckland, Auckland, New Zealand) [22]. Quality control procedures were carried out using PLINK v1.07 (MGH, Boston, MA, USA) to remove SNPs with minor allele frequency (<5%), genotype call rate (<90%), and Hardy–Weinberg equilibrium (*p* < 10^−6^) [23]. Moreover, individuals with more than 10% missing genotypes were removed. Finally, 608,696 autosome SNPs and 1217 individuals remained for the association analyses. The beef cattle datasets are available from the Dryad Digital Repository (doi:10.5061/dryad.4qc06). 

## 3. Results

### 3.1. Simulation Study

#### 3.1.1. Statistical Power for QTN Detection

**Figure 1:**
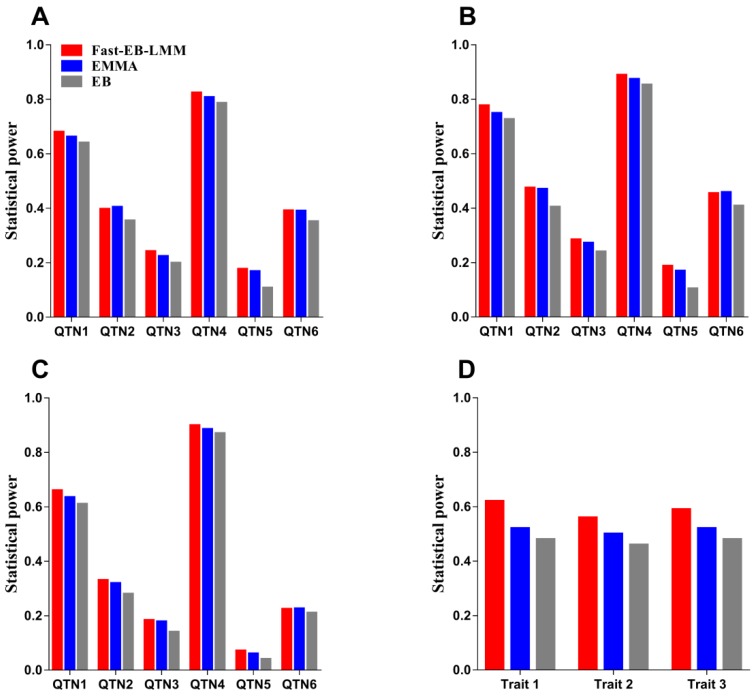
Statistical powers for two different simulation experiments using the three different methods (Fast-EB-LMM, EMMA and EB). The heritabilities were set as 0.10, 0.05, 0.05, 0.15, 0.05 and 0.05 for QTN 1–6 in the first simulation experiment and 0.35, 0.35and 0.50 for Trait 1–3 in the second simulation experiment. (**A**) No polygenic background was simulated in the first simulation experiment. (**B**) A polygenic background was simulated in the first simulation experiment. (**C**) An epistatic background was simulated in the first simulation experiment. (**D**) Statistical power comparison in the second simulation experiment.

Statistical power was used to evaluate the effectiveness of Fast-EB-LMM when compared with the other two methods (EMMA and EB). The statistical power comparison for the two simulation experiments are presented in Figure 1 and Table S1.

For the first simulation experiment, we chose 0.05/m as the designated threshold (Bonferroni correction), where m is the number of SNPs. The statistical power for each QTN was defined as the proportion of samples where the QTN was detected (the *p*-value is smaller than the designated threshold). The ratio of the number of such samples to the total number of replicates (1000) represented the empirical power of this QTN. When only six QTNs were simulated in the first experiment, the statistical powers for QTN detection for Fast-EB-LMM were higher than the other two methods, except QTN2 (Figure 1A; Supplementary Table S1). When a polygenic or epistatic effect was added to the simulated phenotypes, Fast-EB-LMM always had the highest power of detection power for all six QTNs (Figure 1B; Figure 1C). These results demonstrated that Fast-EB-LMM had the highest power over all the three methods under various genetic backgrounds.

For the second simulation experiment, we also set the significance threshold at 0.05/m, which is the same Bonferroni correction as the first simulation experiment. The SNPs whose *p*-value are smaller than the threshold are considered as detected QTNs and a detected SNP within 100 kb of the true QTN was also considered as a true QTN. The statistical power was defined as the total number of true QTNs divided by 50. The results also showed that Fast-EB-LMM always had the highest statistical power for the three simulated traits (Figure 1D).

#### 3.1.2. False Positive Rate and ROC Curve

All the false QTNs, detected by the three methods, in the two simulation experiments were used to calculate the false positive rate of the three methods. These results are listed in Supplementary Table S2 and Table S3. For the first simulation experiment, the three methods had similar false positive rates except that the EB had a higher false positive rate when only six QTNs were simulated. In the second simulation experiments, Fast-EB-LMM had the lowest false positive rate and EB had the highest false-positive rate for all the three simulated traits.

**Figure 2:**
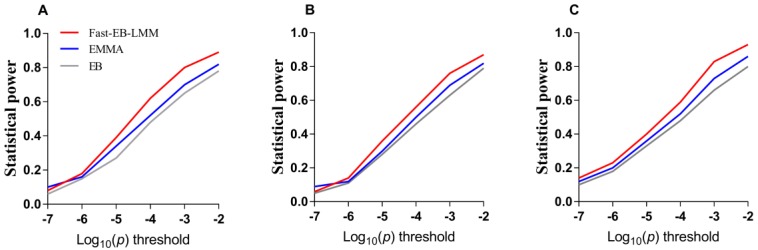
Statistical powers of six simulated QTNs from the first simulation experiment plotted against Type 1 error (in a log10 scale) for the three GWAS methods (Fast-EB-LMM, EMMA and EB). (**A**) No polygenic background. (**B**) With polygenic background. (**C**) With epistatic background.

A receiver operating characteristic (ROC) curve is a plot of the statistical power against the controlled false positive rate. This curve is frequently used to compare different methods for their performance in the detection of significant signals; the higher the curve, the better the method. When six probability levels for significance, from 1E-7–1E-2, were used, the corresponding statistical powers were calculated in the second simulation experiment for the three methods. The results are shown in Figure 2. Clearly, among the three approaches, the Fast-EB-LMM method slightly outperformed the other two methods.

#### 3.1.3. Computational Efficiency

Table 2 shows the computational times of the three methods for GWAS in the second simulation experiment. The EMMA method required 18.85 minutes per trait to complete the analyses, while the Fast-EB-LMM and EB only required 6.01 and 8.79 minutes per trait to complete this process. The Fast-EB-LMM method had relatively high computational efficiency. All three methods were implemented in R (x64 3.4.4) (R Core Team, Vienna, Austria) [24]. Computations were performed on an HP HSTNS-2145 server (Intel Xeon E5-2640 @ 2.60 GHz) with 64.00GB of RAM and the Windows Server 2008 R2 Enterprise operating system (Hewlett-Packard, CA, USA).

### 3.2. Beef Cattle Data

**Figure 3:**
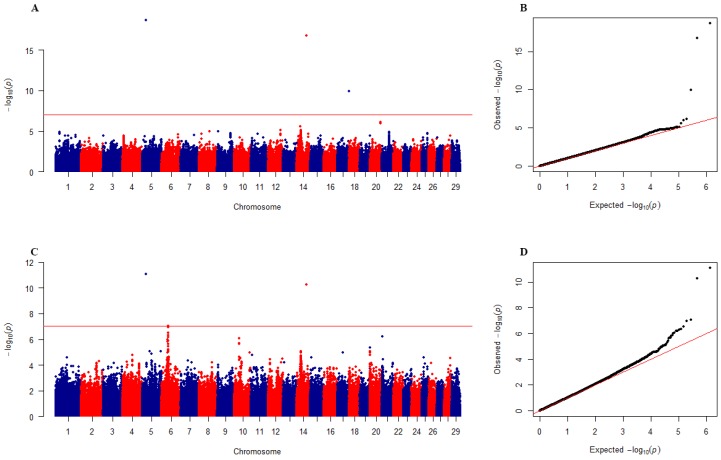
Manhattan and quantile-quantile (QQ) plots of genome-wide association studies for CW and BW in the Chinese Simmental beef cattle population. (**A**,**B**) are Manhattan and QQ plots for CW; (**C**,**D**) are Manhattan and QQ plots for BW.

We first performed a principal component analysis (PCA) using all SNPs and selected the first five principal components as covariates (fixed effects) to control the population structure effects. The fixed effects in the model also included the effects of gender, year, farm, age at slaughter, fattening days and body weights. The modified kinship matrix *K*′ mentioned above was used to account for polygenic background in the model. The Fast-EB-LMM method was used to analyze the beef cattle data for carcass weight (CW) and bone weight (BW) and the results are listed in Table 3. Three SNPs were significantly associated with CW, which include BovineHD0500006528 on BTA 5, BovineHD1400017455 on BTA 14 and BovineHD1700021340 on BTA 17. According the three significant SNPs, we found three candidate genes for CW, which were *C12ORF74, RIMS2 and BT.88981*. Four SNPs weresignificantly associated with BW, which included BovineHD0500006528 on BTA 5, BovineHD0600010952 and BovineHD0600010956 on BTA 6, and BovineHD1400017455 on BTA 14. We also found three candidate genes for BW, which were *C12ORF74, LCORL,* and *RIMS2*. The GWAS results for the two carcass traits are also shown in Figure 3.

## 4. Discussion

Linear mixed model (LMM) has become a widely accepted method in genome wide association studies (GWAS) due to its excellent performance in controlling population structure and cryptic relatedness. However, statistical power and computational efficiency are two major concerns in the LMM-based GWAS methods. Recent study has shown that the SNP being tested and its flanking SNPs should be excluded from the GRM (kinship matrix). If these SNPs are included in the kinship matrix, they will compete with the main effects (as fixed effects) and thus lower the powers of QTN detection. This phenomenon is called “proximal contamination” and is one of the important inducements leading to loss of statistical power for the LMM-based GWAS methods [3,15].

Motivated by the above concerns, several studies have been carried out to address the “proximal contamination” problem. Lippert et al. proposed a Fast-LMM approach using FaST-LMM sampled SNPs uniformly across the genome to construct the GRM and keep the number of SNPs used to create the GRM to be less than the sample size [8]. Meanwhile, a spectral decomposition algorithm was used to speed up matrix operations. The FaST-LMM method produces exactly the same results as a standard LMM, but with a run time and memory footprint far less than the latter [8]. Yang et al. proposed a GCTA-LOCO method, which uses a simpler and more robust strategy than FaST-LMM. When running GCTA-LOCO, all the SNPs on a chromosome where the candidate SNP resides are excluded from the GRM. Studies showed that GCTA-LOCO increased statistical power [16]. However, due to computational time or memory constraints, the routine LMM with the candidate marker included in the kinship matrix is still the most commonly used GWAS method.

In this study, we present a new LMM-based method, called Fast-EB-LMM. We used a strategy that is different from FaST-LMM and GCTA-LOCO by excluding the candidate SNP and its flanking SNPs when constructing the GRM. When SNPs are scanned one at a time, the GRM is always changing. Furthermore, the eigenvalue decomposition and Woodbury matrix identity algorithm were used to ease the computational burden. To validate this method, we performed simulation studies and real beef cattle data analyses.

In the first simulation experiment, there were three different structures of the phenotypic data based on the genotype data of an existing population and three methods (Fast-EB-LMM, EMMA and EB) were used. In the first scenario of the simulation with six simulated QTNs, the Fast-EB-LMM had highest statistical power for all the six QTNs simulated, followed by EMMA and EB. In one occasion (QTN2), the EMMA method was slightly more powerful than the Fast-EB-LMM method (Figure 1A). In the second and third simulation scenarios in which a polygenic or epistatic background was added to the simulation, Fast-EB-LMM was always the most powerful method followed by EMMA and EB (Figure 1B,C). In the second simulation experiment, we further used the 16th QTLMAS workshop dataset to test the validity of Fast-EB-LMM. The results still showed that Fast-EB-LMM outperformed the other two methods (Figure 1D). The ROC curves also reflected the robustness of Fast-EB-LMM (Figure 2). In terms of computational efficiency, by combining the two special computational algorithms, the Fast-EB-LMM method only requires a third of the computing time needed by EMMA and is also faster than EB (see Table 2).

We also applied Fast-EB-LMM to a beef cattle dataset to check for its consistency with previous findings. Two carcass trait including carcass weight and bone weight were analyzed and the results are listed in Table 3. A total of five SNPs and four candidate genes were found to be significantly associated with the two traits. This result is completely consistent with Chang et al. [25]. Moreover, the *LCORL* and *RIMS2* genes found in this study also appeared in Miao et al.’s study [26]. Clearly, Fast-EB-LMM performed well when real data were used.

## 5. Conclusions

In summary, we presented a novel GWAS method that is based on LMM: Fast-EB-LMM. In order to prevent loss of power caused by “proximal contamination”, a modified kinship matrix was used to account for the individual relatedness in the method. The innovation of this modified kinship matrix is that it is constructed by remaining SNPs after exclusion of the candidate SNP and its flanking SNPs. Meanwhile, we used eigenvalue decomposition and Woodbury matrix identity to ease the computational burden. Results from simulation studies show that the Fast-EB-LMM method has the highest power for QTN detection, the highest computational efficiency, and the strongest robustness, compared with the EMMA and EB methods. The beef cattle data analyses also validated the efficiency of the new method. We therefore believe that the Fast-EB-LMM method is a valuable additional tool for GWAS.

## Figures and Tables

**Table 1:** Descriptive statistics of carcass weight (CW) and bone weight (BW) in the Chinese Simmental beef cattle.

Trait	Number	Mean ^a^	SD ^a^	*h* ^2 b^	Phenotypic Correlation	
					CW	BW
CW	1217	271.3	45.63	0.38	1	-
BW	1217	40.7	6.52	0.41	0.67	1

^a^ The unit was kilogram (kg) for CW and BW. ^b^ *h*^2^ was the genomic heritability and evaluated using DMU software (Aarhus University, Aarhus, Denmark).

**Table 2:** Comparison of times taken (Hrs) in the detection of QTNs in the two simulation experiments using the three different methods.

Method	Simulation 1			Simulation 2
	a	b	c	
Fast-EB-LMM	4.17	4.23	4.19	0.1
EMMA	13.82	14.04	13.91	0.31
EB	6.21	6.31	6.24	0.15

a: No polygenic background; b: With polygenic background; c: With epistatic background.

**Table 3:** Significant SNPs and candidate genes identified by the Fast-EB-LMM method for carcass weight (CW) and bone weight (BW) in the Chinese Simmental beef cattle population.

Trait	SNP Name	BTA	Position(bp) ^a^	*p*-Value ^b^	Nearest Gene ^c^	Distance ^d^
CW	BovineHD0500006528	5	22,558,100	2.06E-19	*C12ORF74*	161123
	BovineHD1400017455	14	62769117	1.76E-17	*RIMS2*	within
	BovineHD1700021340	17	73007522	1.12E-10	*BT.88981*	3479
BW	BovineHD0500006528	5	22558100	8.17E-11	*C12ORF74*	161,123
	BovineHD0600010952	6	39990876	8.82E-08	*LCORL*	998873
	BovineHD0600010956	6	39997880	1.13E-07	*LCORL*	1005877
	BovineHD1400017455	14	62769117	5.47E-11	*RIMS2*	within

BTA: Bos Taurus autosome; ^a^ Position(bp) on UMD3.1; ^b^ Genome-wise significance *p*-value after Bonferroni correction. ^c^ Nearest gene found on the Ensembl database(www.ensembl.org). ^d^ Distance between SNP and the nearest gene.

## Supplementary Materials

The following are available online at https://www.mdpi.com/2076-2615/9/6/305/s1, Table S1: Comparison for statistical power in the first simulation experiment using three GWAS methods. Table S2: Comparison for false positive rates in the first simulation experiment using three GWAS methods. Table S3: Comparison for false positive rates in the second simulation experiment using three GWAS methods. File S1: R codes for Fast-EB-LMM.

## Abbreviations

The following abbreviations are used in this manuscript: LMM: Linear mixed model; GWAS: Genome-wide association study; EMMA: efficient mixed model association; GRM: Genetic relationship matrix; SNPs: Single nucleotide polymorphisms; MLMe: Mixed linear model with the candidate marker excluded; QTL: Quantitative trait loci; QTN: Quantitative trait nucleotide; REML: Restricted maximum likelihood; ROC: Receiver operating characteristic; PCA: Principal component analysis; CW: Carcass weight; BW: Bone weight; MME: Mixed model equation; BLUE: Best linear unbiased estimate; EB: empirical Bayes; BTA: Bos taurus autosome.
