# Supplementary material for: A Fast and Powerful Empirical Bayes Method for Genome-Wide Association Studies
Source: Animals (Basel). 2019 May 31;9(6):305. doi: 10.3390/ani9060305 (PMC6616871; doi:10.3390/ani9060305)
Supplement: Supplementary file 1 [file animals-09-00305-s001.zip › Table S1.docx]

Table S1| Comparison for statistical power in the first simulation experiment using three GWAS methods

| **Background** | **Method** | **QTNs** | | | | | |
| --- | --- | --- | --- | --- | --- | --- | --- |
|  |  | **QTN1** | **QTN2** | **QTN3** | **QTN4** | **QTN5** | **QTN6** |
| Six simulated QTNs | Fast-EB-LMM | 0.68 | 0.397 | 0.241 | 0.824 | 0.176 | 0.391 |
|  | EMMA | 0.662 | 0.404 | 0.223 | 0.807 | 0.168 | 0.39 |
|  | EB | 0.64 | 0.354 | 0.199 | 0.786 | 0.107 | 0.351 |
| Six simulated QTNs  + polygenes | Fast-EB-LMM | 0.777 | 0.474 | 0.284 | 0.889 | 0.187 | 0.454 |
|  | EMMA | 0.749 | 0.47 | 0.272 | 0.874 | 0.169 | 0.458 |
|  | EB | 0.726 | 0.404 | 0.24 | 0.853 | 0.104 | 0.408 |
| Six simulated QTNs  + epistatic | Fast-EB-LMM | 0.66 | 0.33 | 0.183 | 0.899 | 0.071 | 0.224 |
|  | EMMA | 0.635 | 0.319 | 0.178 | 0.885 | 0.06 | 0.226 |
|  | EB | 0.61 | 0.28 | 0.14 | 0.87 | 0.04 | 0.21 |
