# Supplementary material for: A Fast and Powerful Empirical Bayes Method for Genome-Wide Association Studies
Source: Animals (Basel). 2019 May 31;9(6):305. doi: 10.3390/ani9060305 (PMC6616871; doi:10.3390/ani9060305)
Supplement: Supplementary file 1 [file animals-09-00305-s001.zip › Table S2.docx]

Table S2| Comparison for false positive rates in the first simulation experiment using three GWAS methods

| **Background Method** | Fast-EB-LMM | EMMA | EB |
| --- | --- | --- | --- |
| Six simulated QTNs | 6.004E-04 | 7.004E-04 | 9.005E-04 |
| Six simulated QTNs  + polygenes | 4.002E-04 | 5.003E-04 | 1.001E-03 |
| Six simulated QTNs  + epistatic | 7.004E-04 | 8.005E-04 | 1.301E-03 |
