# Supplementary material for: A Fast and Powerful Empirical Bayes Method for Genome-Wide Association Studies
Source: Animals (Basel). 2019 May 31;9(6):305. doi: 10.3390/ani9060305 (PMC6616871; doi:10.3390/ani9060305)
Supplement: Supplementary file 1 [file animals-09-00305-s001.zip › Table S3.docx]

Table S3| Comparison for false positive rates in the second simulation experiment using three GWAS methods

| **Trait Method** | Fast-EB-LMM | EMMA | EB |
| --- | --- | --- | --- |
| Trait 1 | 2.31E-03 | 2.11E-03 | 4.12E-03 |
| Trait 2 | 2.01E-03 | 2.21E-03 | 3.12E-03 |
| Trait 3 | 3.02E-03 | 2.91E-03 | 4.72E-03 |
